# Kinetic Modeling of Deep Oxidative Desulfurization over Functionalized UiO-66 from a Model Fuel Using Complex Reaction Theory

**DOI:** 10.1021/acsomega.4c06722

**Published:** 2025-04-15

**Authors:** Bijan Barghi, Tanel Mõistlik, Deniss Panov, Anastassia Raag, Oliver Järvik, Allan Niidu

**Affiliations:** † Virumaa College, School of Engineering, Tallinn University of Technology, Kohtla-Järve 30322, Estonia; ‡ Department of Energy Technology, Tallinn University of Technology, Tallinn 19086, Estonia

## Abstract

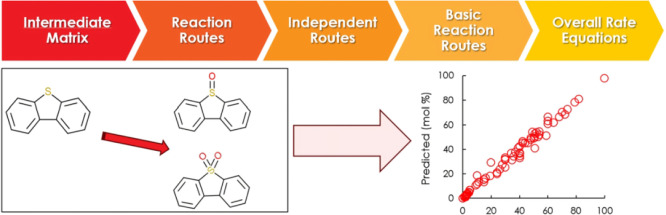

This study proposes a systematic and straightforward approach for determining basic reaction routes and overall reactions using the theory of complex kinetics for an oxidative desulfurization (ODS) reaction using solvothermally synthesized metalorganic framework (MOF), UiO-66-NO_2_ as a catalyst to remove the dibenzothiophene (DBT) from a model fuel in the temperature range of 20–100 °C. The intermediates are organized in a ″matrix of intermediates″ that simplifies the elementary reactions combination to form overall reactions. Adsorption, oxidation, and desorption were taken into consideration in 11 basic reaction steps that comprised the suggested ODS mechanism. The model’s kinetic parameters were optimized by MATLAB program and described by the reparametrized Arrhenius equation. Markov chain Monte Carlo (MCMC) modeling was used to investigate the dependability of the estimated parameters and model predictions. The experimental results were satisfactorily in agreement with the model predictions, as the complex reaction was utilized for the first time to investigate the ODS reaction over MOFs.

## 1 Introduction

The growth of the global population, along with advancements in economic activities, has led to a rise in the demand for fossil fuels across multiple sectors. This includes the need for energy inputs in industrial sectors.^1−6^ Various fuels, such as diesel and gasoline, which are produced from crude oil, contain different sulfur-containing compounds, such as thiophenes. The combustion of fossil fuels results in the release of sulfur oxides, causing environmentally harmful pollution.^7,8^ The hydrodesulfurization process, which uses chromium/molybdenum catalysts, is the conventional technology for removing organic sulfur from liquid fuels; however, as a byproduct, this process produces undesired H_2_S gas. Nevertheless, this method is only financially viable when eliminating specific sulfur compounds, such as dibenzothiophene.^9^ The approach is not capable of entirely removing aromatic sulfur-containing compounds. To address this issue, alternative methodologies e.g., ODS, selective adsorption,^9,10^ and biodesulfurization^11^ have been studied. One of the techniques mentioned, ODS, is acknowledged as a green approach for the desulphurization of benzothiophene derivatives.^12^ It has gained considerable attention because of its simple and straightforward operating conditions and its positive impact on the economy and the environment.^13^

Metal–organic frameworks (MOFs) have gained significant attention due to their unique properties that could be tailored according to specific needs.^14^ These materials consist of metal centers bonded to organic ligands, resulting in a flexible structure with chemical tunability, high specific surface area, and high porosity.^15^ Due to their tunability and diverse chemical compositions, MOFs find applications in various fields.^16−21^ ZrO_2_ forms Zr_6_ clusters (Zr_6_O_4_(OH)_4_) composed of six zirconium atoms arranged in an octahedral geometry, which are interconnected by eight μ_3_-oxygen atoms.^22^ These clusters can be reversibly reconfigured without breaking connecting oxygen bridges leading to extraordinary robustness of Zr-based MOFs.^22^ For instance, UiO-66(Zr) as a zirconium-based MOF, where the hexanuclear zirconium clusters are connected to terephthalic acid (H_2_BDC) organic linkers,^23,24^ exhibits high thermal stability.

Different functional organic linkers in UiO-66 have been reported to offer a strong affinity toward various environmental contaminants and to have the potential to significantly impact chemical activity.^25−27^ Nitro-modified UiO-66 (UiO-66-NO_2_) exhibited high catalytic activity in the deep ODS due to the more electron-accepting group in its ligand structure.^28,29^ Recent advancements have led to the development of MOFs with enhanced stability against oxidants like H_2_O_2_ and oxygen, addressing previous concerns with prior MOFs.^30,31^ Because of its high effective oxygen content, H_2_O_2_ has been used extensively as an oxidizing agent during oxidative desulfurization producing water, as a byproduct,^32^ an environmentally benign compound, making it an oxidant of choice in the current work. The oxidation of dibenzothiophene (DBT) proceeds through two primary stages, first yielding dibenzothiophene sulfoxide (DBTO) and then dibenzothiophene sulfone (DBTO_2_). DBTO, also known as mono-oxy DBT or sulfoxide, is formed when a single oxygen atom is added to the sulfur atom in the DBT molecule, representing the initial step in the oxidation process. Further oxidation results in DBTO_2_, or dioxygen DBT, where two oxygen atoms are attached to the sulfur atom, marking the final stage of this oxidation pathway.

It is evident that ODS stands out as a highly promising technique for fuel desulfurization.^9−11^ While numerous studies have reported catalytic data on various catalysts in sulfur oxidation reactions, there is a significant scarcity of a proper kinetic study.^33−37^ Most studies contained a kinetic modeling with a first-order kinetic study^33−36^ and kinetics employing Langmuir–Hinshelwood and Eley–Rideal mechanisms.^37^ Complex reaction methodology^38^ employs reaction route networks to construct a kinetic approach for the mechanisms through an intermediate matrix.^3,4^ The intermediate matrix represents the stoichiometric relationships between chemical species and elementary reactions.^38^ ODS illustrates a reaction network including multiple reactions, which made it necessary to develop a practical procedure for calculating the rate of complex multistep reactions. Previous studies discussed the kinetics of the ODS; however, oxidation products were not reported.^39,40^ The theory of complex reactions allows for a more detailed understanding of multistep reaction mechanisms rather than relying solely on the concept of a single rate-determining step. The idea of a rate-determining step (RDS) procedure is not suitable for the reactions with multiple steps, reported before the ODS case.^41^ Instead of focusing on a single RDS that can oversimplify complex mechanisms, complex reaction theory considers the interplay between different steps and how they contribute to the overall reaction rate. Researchers have invested significant effort in developing a systematic approach to address complex reaction networks. When reaction mechanisms are described, stoichiometric relationships between the chemical species involved are essential, particularly in complex reactions. Horiuti and Nakamura^42^ formulated the comprehensive theory of stoichiometric numbers for elementary reactions, proposing the concept of overall reactions. Following that, Horiuti and Nakamura^43^ and Temkin^44^ introduced the theory of complex reactions, which facilitated the development of a kinetic model for multiple mechanisms,^45^ particularly on the catalyst surface, as it provided an efficient algorithm for estimating the rates of complex multistep reactions. In such procedures that Murzin reported, a singular rate-limiting step may be absent, and the prevalent practice of incorporating such stages primarily results from the convenience of using simpler expressions.^46^ Markov chain Monte Carlo (MCMC) method is employed to model the kinetics of reactions accompanied by parameters.^47^ MCMC provides a complete picture of parameter uncertainties and their dependencies, which is a core aspect of Bayesian inference. This methodology is applicable for the case with chemical kinetics models that are typically described by systems of differential equations representing reaction rates and species concentrations, in various kinetic study cases, cell culture process in a bioreactor,^47^ isobutane dehydrogenation,^48^ catalytic oxidation,^49^ and lactose hydrogenation over ruthenium.^50^

Previously, to the best of our knowledge, no report has been presented describing a straightforward mathematical procedure for ODS to obtain the overall rate reaction of a standard reactant/product involved in the elementary steps based on the theory of complex reactions. The aim of this study is to create a comprehensive kinetic model using the complex reaction methodology to analyze the ODS mechanism. The dependability of the estimated parameters and model predictions is investigated by the application of Markov chain Monte Carlo (MCMC) techniques.

## 2 Experimental Section

### 2.1 Materials

Zirconium(IV) chloride (ZrCl_4_, 98%, Acros), nitroterephthalic acid (99%, Acros), hydrochloric acid (HCl, 36%, Honeywell), *N*,*N*-dimethylformamide (DMF, 99.5%, Fisher), ethanol (C_2_H_5_OH, 99.9%, Honeywell), acetonitrile (99.9%, Honeywell), n-dodecane (99%, Alfa Aesar), hydrogen peroxide (H_2_O_2_, 30%, Alfa Aesar), and dibenzothiophene (98%, Acros) were used without any further purification.

### 2.2 Synthesis of MOF

MOFs were synthesized as originally described.^51^ 1 g of ZrCl_4_ and 1.25 g of nitro terephthalic acid were dissolved in 120 mL of DMF and 8 mL of 36% and then sonicated for 35 min. In an oven, the prepared solution was heated at 80 °C for 24 h. Upon cooling to room temperature, the sample was washed using DMF and ethanol and dried by heating under 600 mbar pressure at 80 °C on a rotary evaporator. The characterization of the synthesized UiO-66-NO_2_ MOF was previously reported.^52^

### 2.3 Oxidative Desulfurization Test

ODS reactions were conducted in a 22 mL glass vial reactor. For the model fuel (MF), 1000 ppm of DBT was dissolved in n-dodecane (3 mL). To this solution in the reactor, acetonitrile (3 mL) was added as the polar phase in a 1:1 ratio by volume (3 mL of MF and 3 mL of acetonitrile). Upon oxidation, the polarity of sulfur-containing compounds is increased, enabling their extraction (DBTO and DBTO_2_) into acetonitrile as the polar phase. 62 mg of UiO-66-NO_2_ was added to the reactor; then 13.1 mL of hydrogen peroxide (aq. 30%) was added after heating the reactor to a predefined temperature (at 20, 36, 60, 84, and 100 °C) under atmospheric pressure. The polar and fuel phases were separated after the reaction had completed (60 min) and then analyzed by a Shimadzu QP2010 Plus GC to measure the sulfur compounds. The DBT conversion, *X*_DBT_ [mol %], selectivity, *S*_*i*_ [mol %], and product (DBTO and DBTO_2_) yield, *Y*_*i*_ [mol %], were calculated by the following eqs 1^3^):
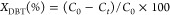
1
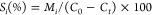
2
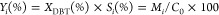
3

*C*_0_ [mol.m^–3^] and *C*_*t*_ [mol.m^–3^] correspond to the DBT concentration in MF at the beginning and end of the reaction, respectively, and *M*_*i*_ [mol.m^–3^] denotes the concentration of component *i*.

### 2.4 Kinetic Study

The ODS mechanism was presented, along with the number of monomolecular and bimolecular reactions. The product stream constitutes of water, mono-oxy DBT (DBTO or sulfoxide), dioxygen DBT (DBTO_2_ or sulfone), and DBT. The proposed ODS mechanism of DBT over MOF involves 11 elementary steps illustrating the production of primary sulfone (dioxygen sulfur) and sulfoxide (mono-oxy sulfur) in subsequent reaction stages:

Adsorption of the reactant and oxidant:
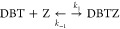
4
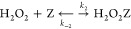
5

Surface reactions:
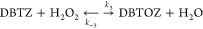
6
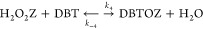
7
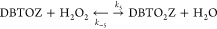
8
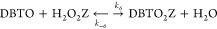
9
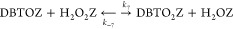
10
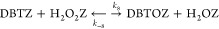
11

Desorption:
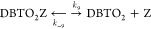
12
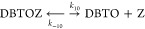
13
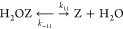
14

It is noted that Z is the MOF metal node catalytic site, and RZ (R: reactant/products) denote different surface species, also *k*_*i*_ is the intrinsic kinetic constant of elementary step *i.*Table 1 displays 11 surface reactions for the investigation of reaction kinetics. Additionally, Table 2 illustrates the matrix of surface species in the DBT oxidative desulfurization steps. The rate equations were formulated according to the concentration of the reaction participants.

**Table 1:** Rate Equations of Elementary Surface Reactions Proposed for ODS

Elementary reaction route	Rate equations	Rate constant unit
(1)	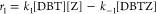	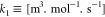
		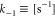
(2)	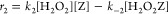	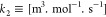
		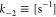
(3)	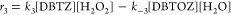	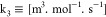
		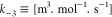
(4)	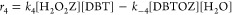	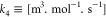
		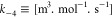
(5)	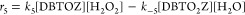	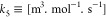
		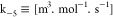
(6)	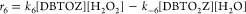	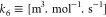
		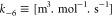
(7)	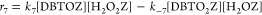	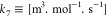
		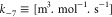
(8)	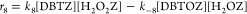	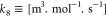
		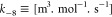
(9)	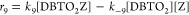	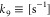
		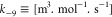
(10)	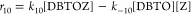	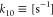
		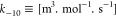
(11)	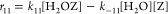	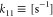
		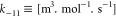

**Table 2:** Matrix of Surface Species in DBT ODS Reaction Steps

Reaction no.	Reactions	Z	DBTZ	DBTOZ	DBTO_2_Z	H_2_O_2_Z	H_2_OZ
1	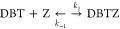	–1	+1	0	0	0	0
2	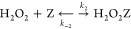	–1	0	0	0	+1	0
3	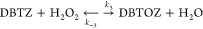	0	–1	+1	0	0	0
4	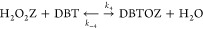	0	0	+1	0	–1	0
5	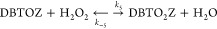	0	0	–1	+1	0	0
6	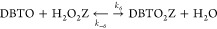	0	0	0	+1	–1	0
7	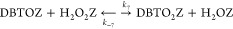	0	0	–1	+1	–1	+1
8	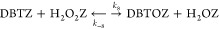	0	–1	+1	0	–1	+1
9	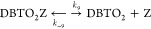	–1	0	0	+1	0	0
10	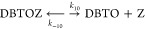	–1	0	+1	0	0	0
11	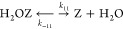	–1	0	0	0	0	+1

For the extraction oxidation of DBT, an irreversible first-order reaction is considered.^53^ The lump kinetic model equations in the final section of the ODS can be obtained by deriving them from the continuity equation and reaction rate equation, assuming:
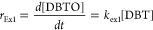
15
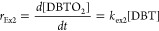
16

The present study considers the comprehensive scenario of a chemical reaction system, which is explained by a reaction mechanism comprising ρ elementary reaction steps *s*_ρ_ (ρ = 1,2,···,*p*). The components participating in these elementary reaction steps are categorized into *k* = 1 to *l* intermediates, referred to as surface species, labeled as *I*_1_, *I*_2_, *I*_3_, and so forth, and *n* molecular reactants/products, represented as *T*_1_, *T*_2_, *T*_3_, and so forth. It should be noted that elementary reaction steps can be denoted as follows:^3^
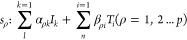
17

It is widely acknowledged that the stoichiometric coefficients of the molecular reactants/products β_ρ*i*_ and the intermediates α_ρ*k*_ possess negative values for reactants and positive values for products. The combination of stages is employed to eliminate the intermediates and achieve the formation of overall reactions. To accomplish this, a procedure is established that relies on the surface species. During the initial phase, the intermediates are collected in a separate matrix called ″intermediates matrix″.^4^ The stoichiometry and intermediate matrix has been proposed as a tool to directly determine the structure of reaction routes in Bray–Liebhafsky reaction,^54^ homogeneous organic reaction system,^55^ liquified petroleum gas catalytic cracking,^3,4^ Prins condensation of isopulegol over zeolite,^56^ and the other studies.^38,57−59^

Disregarding the second term within the expression (17) and focusing solely on , we can represent the intermediates and their respective stoichiometric coefficients in a three-dimensional matrix referred to as *IM*(*s*_ρ_,*I*_*l*_,α_ρ*l*_). This matrix is structured such that the primary dimension, *s*_ρ_, delineates a reaction step or stage ρ, the secondary dimension, *I*_*l*_, signifies an intermediate involved in the reaction. Lastly, the third dimension, α_ρ*l*_, denotes the corresponding stoichiometric coefficient of *I*_*l*_ in the reaction step *s*_ρ_. The presence of an intermediate within a particular reaction stage *s*_ρ_ is denoted by a value of 1 in the matrix, whereas its absence is represented by a value of 0, also for all molecules (reactant and product molecules, e.g., DBT) the coefficient value is considered zero.

Ultimately, the intermediate matrix (Table 3) undergoes a rearrangement process utilizing the sparse equation solving method due to the prevalent presence of numerous zero elements within the intermediate matrix. The intermediates with higher frequencies are shifted to the former columns and rows. Supposing that the most repeated intermediate (MRI) appears 11 times in the mechanism (surface reaction steps). Consequently, the first column in the table is dedicated to the MRI, and the primary rows represent the stages that involve this intermediate.

**Table 3:** Rearranged Matrix for Elementary Steps of DBT Oxidative Desulfurization

Reaction no.	Reactions	Z	DBTZ	DBTOZ	DBTO_2_Z	H_2_O_2_Z	H_2_OZ
1	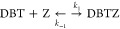	–1	+1	0	0	0	0
10	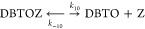	–1	0	+1	0	0	0
9	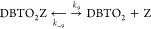	–1	0	0	+1	0	0
2	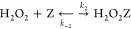	–1	0	0	0	+1	0
11	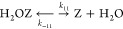	–1	0	0	0	0	+1
3	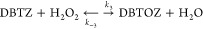	0	–1	+1	0	0	0
8	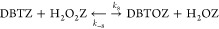	0	–1	+1	0	–1	+1
5	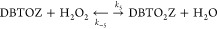	0	0	–1	+1	0	0
7	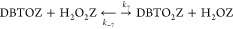	0	0	–1	+1	–1	+1
4	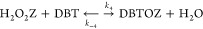	0	0	+1	0	–1	0
6	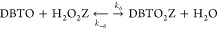	0	0	0	+1	–1	0

Overall reactions are the linear combinations of surface reaction steps produced by multiplying reaction equations of stages by stoichiometric coefficients; these coefficients have to be selected so that there are no surface species in the overall reaction. A reaction route consists of a set of stoichiometric steps and must be unique. However, individual overall reactions can be the same, and it is impossible to derive a single reaction route by multiplying another route by a number. *P*, the number of entire reaction pathways, is ascertained as follows.^3,4^
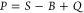
18

The variables *S*, *B*, and *Q* represent the elementary step number, the number of governing equations between adsorbed intermediates, and the intermediate number, respectively. Six fundamental pathways are anticipated due to the proposed mechanism’s 11 steps, six surface species (intermediates), and one equation between adsorbed intermediate (eq 39). At this point, the user identifies the reaction routes on the basis of these routes, as shown in Table 4.

**Table 4:** Reaction Routes and Overall Reactions and Basis of Reaction Routes

Reaction route	Expression	Overall reaction	
1	S1–S3+S10	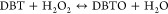	N^(1)^
2	S9–S5+S10	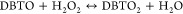	N^(2)^
3	S4–S9+S10-S6	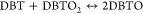	N^(3)^
4	S3–S5+S7-S8	-	N^(4)^
5	S2+S4+S10	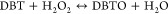	N^(5)^
6	S11+S7–S5+S2	-	N^(6)^

The basic routes were used to derive the stoichiometric matrix of reaction routes, *n*, given in Table 5.

**Table 5:** Stoichiometric Matrix of Reaction Routes

No.	*N*^(1)^	*N*^(2)^	*N*^(3)^	*N*^(4)^	*N*^(5)^	*N*^(6)^
1	+1	0	0	0	0	0
2	0	0	0	0	+1	+1
3	–1	0	0	+1	0	0
4	0	0	+1	0	+1	0
5	0	–1	0	–1	0	–1
6	0	0	–1	0	0	0
7	0	0	0	+1	0	+1
8	0	0	0	–1	0	0
9	0	+1	–1	0	0	0
10	+1	+1	+1	0	+1	0
11	0	0	0	0	0	+1

The concentration of an intermediate remains constant over time due to the equilibrium between the rates of formation and consumption in different elementary reactions. Each pathway can be expressed as a linear combination of its basic routes. The rate for basic route *N*^(*P*)^ (as shown in Table 5) is considered *r*^(*P*)^, according to its definition, there are  runs of the *S*^th^ stage.  is the stoichiometric number of the *S*^th^ step along the route *N*^(*P*)^, *r*_*s*_ and *r*_*–s*_ are the rates of the forward and reverse elementary reactions comprising the “*S*” stage. The total number of runs of *S*^th^ is obtained by summation over all “*P*” basic routes (eq 18). The relationship between the rate of elementary steps and the reaction routes was explained as :
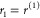
19
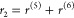
20
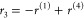
21
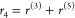
22
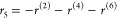
23
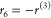
24
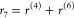
25
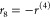
26
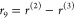
27
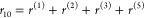
28
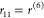
29

A quasi-steady-state governing system is thought to exist for this set of reactions. The following relations will be present in the system of equations that the stoichiometric matrix generates:
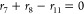
30
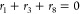
31
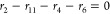
32
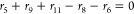
33
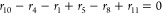
34

By providing the individual rates of reaction steps following the mass action law, we can derive the following mathematical eq 35^39^ and balance eq 40:
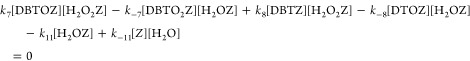
35
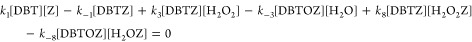
36
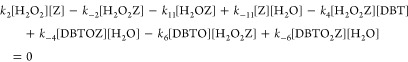
37
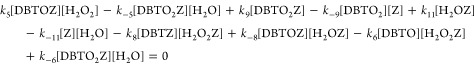
38


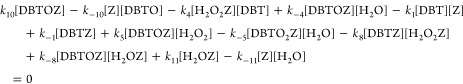
39


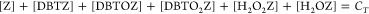
40

eq 40 presents the site balance equation, indicating that the sum of all intermediates and free sites is equal to the total number of sites, *C*_*T*_. At this point, the equations below can be used to derive the relationship between the reaction stages and the formation of compounds involved.
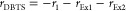
41
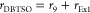
42
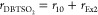
43

### 2.5 Modeling and Parameter Estimation

The kinetic study was performed on UiO-66-NO_2_ using a complex reaction methodology. The kinetic rate parameters were calculated by minimizing the objective function that compares the model predicted values with the experimental data. The determination of kinetic coefficients in heterogeneous catalytic reactions contains the analysis of nonlinear equations to assess the kinetic study. In this study, the governing differential equation for a batch reactor is presented in eq 44:
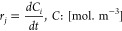
44
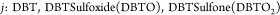


The molar production rate of component *j*, denoted as *r*_*j*_, is determined by the rate equations of reaction steps; *C*_*i*_ is the concentration of species *i.* The system eqs (eqs 40, 42, and 43) were solved simultaneously using the ODE45 function in MATLAB programming software to obtain the yields, a function used for solving differential equations. ODE45 is appropriate mainly for solving first-order equations that are time-dependent.^60^ The optimum parameter estimation was obtained using the experimental species yield data by minimizing the root-mean-square error (RMSE) for the component yield defined by eq 45.
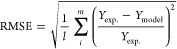
45

Here *l* is the experimental point numbers, *m* is the number of experimental points at each temperature setting, and *Y*_*i*_ is the component yield in an experiment and predicted by the model. The optimization process utilized the genetic algorithm toolbox provided by MATLAB. The activation energy and pre-exponential factor of reactions were tracked through the reparameterized Arrhenius equation in terms of kinetic parameters, as follows:^61^


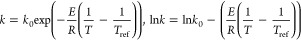
46

The reaction rate constant, denoted as *k*, pre-exponential factor, denoted as *k*_0_, activation energy *E* (kJ mol^–1^), denoted as universal gas constant *R* (kJ mol^–1.^K^–1^), and the reaction temperature, denoted as *T*(K) and *T*_ref_ (K), 298.15.

## 3 Results and Discussion

### 3.1 Effect of Temperature over ODS

**Figure 1:**
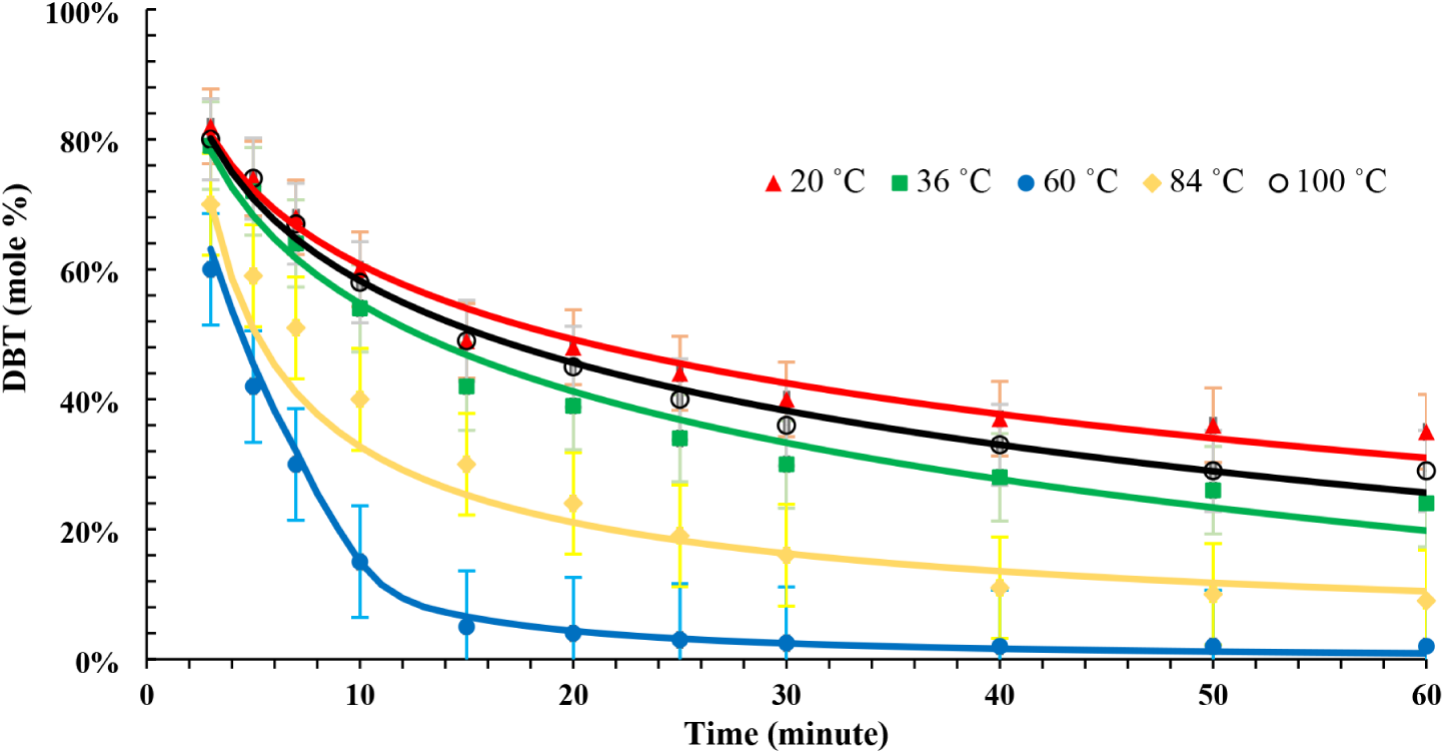
DBT (mol %) for the ODS reaction (pressure: atmospheric, temperatures: 20, 36, 60, 84, and 100 °C) “experimental data: dots, predicted results: lines”.

The reactions were conducted at five temperatures in 1 h. Experimental data and the model predicted DBT mole fraction are demonstrated in Figure 1 and reported in Table 6. The dibenzothiophene oxidation yield is increased by raising the reaction temperature from 20 °C. However, the efficiency of dibenzothiophene oxidation decreases when the temperature exceeds an optimum temperature.^52^ Since the oxidative desulfurization reaction is endothermic, raising the temperature promotes the conversion rate of DBT and enhances the molecular mobility of the reaction components. Nevertheless, raising the temperature causes the decomposition of hydrogen peroxide, and therefore, the rate of the ODS reaction decreases as the concentration of the oxidant is reduced.^52^

**Table 6:** Experimental Results and Predicted Model Data of DBT (Mol %) for Oxidative Desulfurization Reaction in Atmospheric Pressure and Different Temperatures^a^

	20 °C		36 °C		60 °C		84 °C		100 °C	
Time (min)	Exp.	Pred.	Exp.	Pred.	Exp.	Pred.	Exp.	Pred.	Exp.	Pred.
5	74.2%	(72.2 ± 3.0)%	72.0%	(68.2 ± 3.5)%	42.1%	(45.4 ± 3.1)%	59.4%	(50.8 ± 2.7)%	74.3%	(70.8 ± 2 .1)%
10	60.3%	(60.7 ± 2.5)%	54.1%	(54.7 ± 3.0)%	15.1%	(15.0 ± 2.5)%	40.4%	(32.7 ± 2.2)%	58.2%	(58.2±0.7)%
15	49.2%	(54.0 ± 2.3)%	42.3%	(46.8 ± 2.7)%	5.2%	(6.6 ± 2.1)%	30.3%	(25.3 ± 1.9)%	49.3%	(50.9±0.3)%
20	48.0%	(49.2 ± 2.1)%	39.2%	(41.2 ± 2.4)%	4.3%	(4.4 ± 1.9)%	24.6%	(21.1 ± 1.7)%	45.1%	(45.6±0.2)%
25	44.0%	(45.5 ±1.9)%	34.4%	(36.9 ± 2.2)%	3.4%	(3.2 ± 1.7)%	19.1%	(18.3 ± 1.5)%	40.0%	(41.6±0.2)%
30	40.1%	(42.5 ± 1.8)%	30.6%	(33.3 ± 2.1)%	2.5%	(2.4 ± 1.5)%	16.1%	(16.3 ± 1.3)%	36.2%	(38.2 ±0.1)%
40	37.4%	(37.7 ± 1.6)%	28.1%	(27.7 ± 1.9)%	2.4%	(1.6 ± 1.3)%	11.1%	(13.5 ± 1.1)%	33.1%	(33.0 ±0.1)%
50	36.0%	(34.0 ± 1.4)%	26.0%	(23.3 ± 1.7)%	2.1%	(1.2 ± 1.1)%	10.4%	(11.8 ± 0.9)%	29.3%	(28.9 ± 0.1)%
60	35.2%	(31.0 ± 1.3)%	24.3%	(19.8 ± 1.5)%	2.0%	(0.9 ± 0.9)%	9.3%	(10.5 ± 0.8)%	29.1%	(25.6 ± 0.0)%

a Standard uncertainties (*U*) are *U* (*T*) = ± 1 °C; *U* (time) = ± 1 min

**Figure 2:**
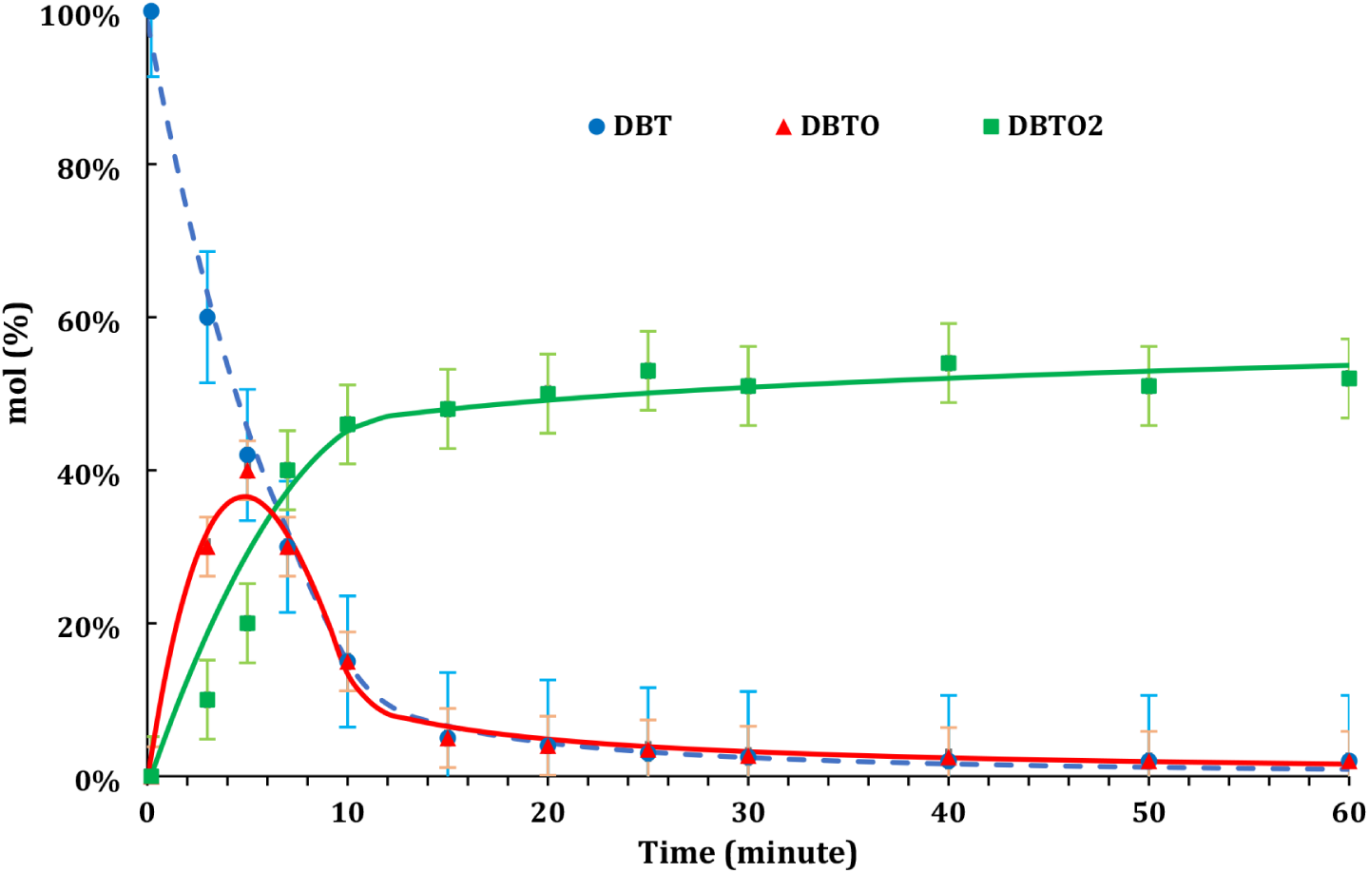
Yield (mol %) of DBTO, DBTO_2_, and DBT mole fraction (mol %) for the ODS reaction (pressure: atmospheric, temperature: 60 °C) “experimental data: dots, predicted results: lines”.

The component yields obtained from the experiment and the calculations are illustrated in Figure 2. The product distribution results showed that the DBT oxidized to sulfoxide (DBTO) and sulfone (DBTO_2_) in the presence of UiO-66-NO_2_. The experimental and kinetic predicted results for the DBT mole fraction and oxidized products yield are reported in Table 7.

**Table 7:** Experimental Results and Predicted Model Data of DBT, DBTO, and DBTO_2_ Yield (mol %) Oxidative Desulfurization of the Model Fuel over UiO-66-NO_2_ at Temperature: 60 °C^a^

	DBT		DBTO		DBTO_2_	
Time	Exp.	Pred.	Exp.	Pred.	Exp.	Pred.
0	100%	(98.7 ± 6.3)%	0	(0.3 ± 0.0)%	0	(1.4 ± 0.1)%
5	42.0%	(45.4 ± 4.3)%	40.0%	(36.5 ± 1.2)%	20.7%	(29.1 ± 1.4)%
10	15.4%	(15.0 ± 1.4)%	15.3%	(13.4 ± 0.4)%	46.0%	(45.1 ± 2.2)%
15	5.5%	(6.6 ± 0.6)%	5.0%	(6.5 ± 0.2)%	48.1%	(48.0 ± 2.4)%
20	4.4%	(4.4 ± 0.4)%	4.3%	(4.9 ± 0.2)%	50.1%	(49.2 ± 2.4)%
25	3.2%	(3.2 ± 0.3)%	3.5%	(3.9 ± 0.1)%	53.3%	(50.1 ± 2.5)%
30	2.5%	(2.4 ± 0.2)%	2.7%	(3.2 ± 0.1)%	51.1%	(50.8 ± 2.5)%
40	2.4%	(1.6 ± 0.2)%	2.5%	(2.4 ± 0.1)%	54.2%	(52.0 ± 2.6)%
50	2.2%	(1.2 ± 0.1)%	2.3%	(1.9 ± 0.1)%	51.3%	(52.9 ± 2.6)%
60	2.1%	(0.9 ± 0.1)%	2.0%	(1.6 ± 0.1)%	52.1%	(53.7 ± 2.6)%

a Standard uncertainties (*U*) are *U* (*T*) = ± 1 °C; *U* (time) = ± 1 min

**Figure 3:**
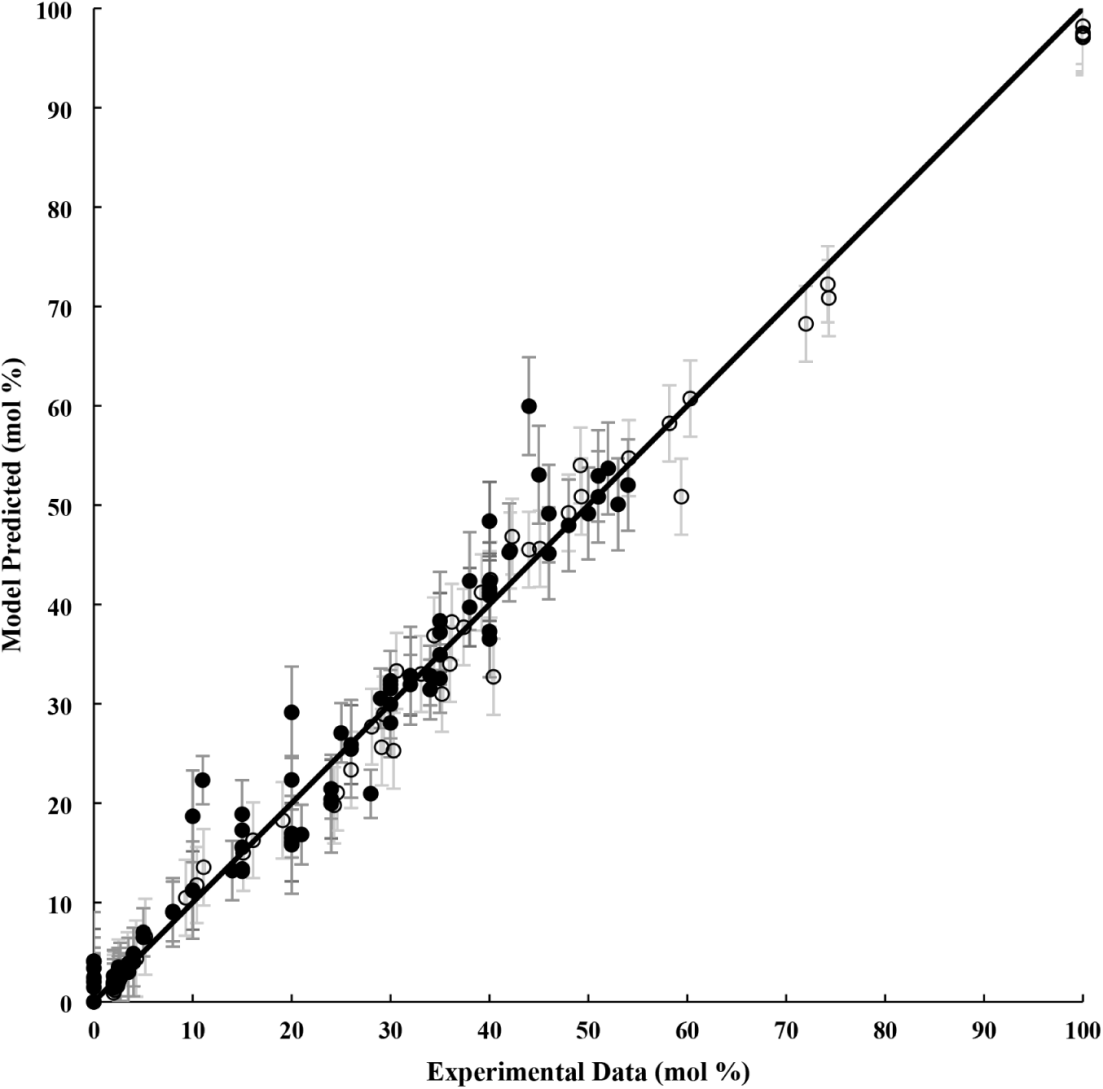
Comparison between experimental data and predicted product yields (mol %) “filled circle” and DBT mole fraction (mol %) “unfilled circle” for the ODS at different temperatures 20, 36, 60, 84, 100 °C.

A parity plot in Figure 3 demonstrates satisfactory agreement between the experimental and predicted values across the various operating conditions investigated in this study. The optimal kinetic parameters were determined with an RMSE value of 10.5%.

### 3.2 Estimation of Kinetic Parameters

Table 8 presents the results of kinetic optimization, showcasing the activation energy and pre-exponential factor. These results were derived by plotting a linear trendline of ln(*k*_*i*_) against 1/*T* (1/*K*). The slope of the trendline determined the activation energy, and the intercept provided the expression “” for the pre-exponential factor “*k*_0_” calculation. The results depict that five elementary and one lumped reaction illustrate higher kinetic rates, including DBT adsorption (reaction 1), first DBT oxidation (reaction 3), second DBT oxidation (reaction 5), DBTO desorption (reaction 9), DBTO_2_ desorption (reaction 10), and DBTO_2_ extraction to the polar phase (eq 15) which contained three monomolecular reactions, two bimolecular reactions, and one extraction lumped. First, DBT oxidation (reaction step 5) is 1.5 times higher than second DBT oxidation (reaction step 3); also, DBT adsorption is (reaction step 1) 1.8 times higher than second DBT oxidation. Moreover, the DBT adsorption kinetic is greater than DBTO_2_ desorption. The other stages of reactions were disregarded from the reaction mechanism, while maintaining a high level of precision. It is noted that for the mentioned routes, the reversible reactions were not significant and were ignored. Kinetic parameters were obtained while maintaining the absence of the MOF deactivation.

**Table 8:** Optimized Kinetic Parameters, Including Activation Energy and Pre-Exponential Factor for the Proposed Model of DBT Oxidation

Item	Unit	Value	Item	Value (kJ mol^*–*1^)	*R*^2^
*k*_01_	(m^3^.mol^–1^.s^*–*1^)	7.48 × 10^3^	***E*_1_**	13.1	0.954
*k*_02_	(m^3^.mol^–1^.s^*–*1^)	1.84 × 10^2^	***E*_2_**	16.3	0.939
*k*_03_	(m^3^.mol^–1^.s^*–*1^)	7.96 × 10^2^	***E*_3_**	15.1	0.921
*k*_04_	(m^3^.mol^–1^.s^*–*1^)	2.53 × 10^2^	***E*_4_**	14.3	0.993
*k*_05_	(m^3^.mol^–1^.s^–1^)	1.82 × 10^4^	***E*_5_**	16.1	0.981
*k*_06_	(m^3^.mol^–1^.s^*–*1^)	3.04 × 10^2^	***E*_6_**	20.3	0.916
*k*_07_	(m^3^.mol^–1^.s^–1^)	2.08 × 10^2^	***E*_7_**	16.9	0.947
*k*_08_	(m^3^.mol^–1^.s^–1^)	2.84 × 10	***E*_8_**	12.5	0.912
*k*_09_	(s^–1^)	1.48 × 10^4^	***E*_9_**	21.4	0.939
*k*_010_	(s^–1^)	7.80 × 10^3^	***E*_10_**	20.1	0.930
*k*_011_	(s^–1^)	2.19 × 10^2^	***E*_11_**	18.5	0.951
*k*_0–1_	(s^–1^)	1.10 × 10	***E*_–1_**	91.7	0.976
*k*_0–2_	(s^–1^)	7.94 × 10	***E*_–2_**	92.4	0.966
*k*_0–3_	(m^3^.mol^–1^.s^*–*1^)	5.64 × 10	***E*_–3_**	95.3	0.955
*k*_0–4_	(m^3^.mol^–1^.s^–1^)	8.42 × 10^2^	***E*_–4_**	75.3	0.965
*k*_0–5_	(m^3^.mol^–1^.s^–1^)	1.82	***E*_–5_**	37.3	0.952
*k*_0–6_	(m^3^.mol^–1^.s^–1^)	5.64 × 10	***E*_–6_**	89.1	0.953
*k*_0–7_	(m^3^.mol^–1^.s^*–*1^)	3.19 × 10	***E*_–7_**	58.7	0.963
*k*_0–8_	(m^3^.mol^–1^.s^–1^)	2.91	***E*_–8_**	46.6	0.925
*k*_0–9_	(m^3^.mol^–1^.s^–1^)	3.87	***E*_–9_**	55.3	0.944
*k*_0–10_	(m^3^.mol^–1^.s*^–^*^1^)	3.50	***E*_–10_**	61.7	0.938
*k*_0–11_	(m^3^.mol^–1^.s^*–*1^)	3.74	***E*_–11_**	51.1	0.933
*k*_0ex1_	(s^–1^)	1.87 × 10^3^	***E*_ex1_**	33.7	0.929
*k*_0ex2_	(s^–1^)	1.70 × 10^–2^	***E*_ex2_**	31.3	0.954

### 3.3 MCMC Analysis

**Figure 4:**
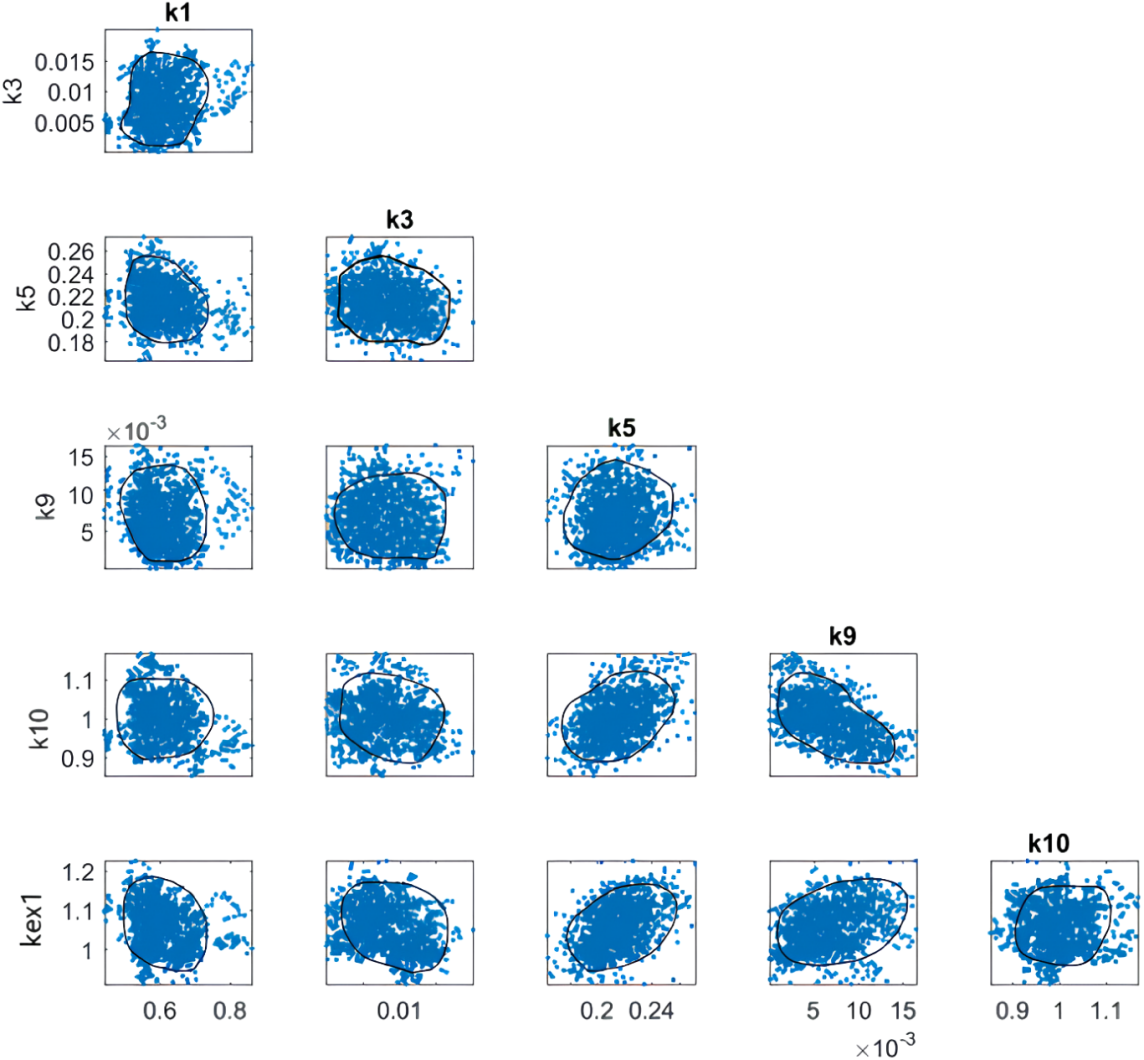
Correlation matrix of the parameters using the MCMC method (the density of the dots in each subfigure represents probability, and the circles represent 95% probability regions of the parameter).

The dependability of the six main reaction kinetics was assessed by Markov chain Monte Carlo (MCMC). The findings from the MCMC technique, which employs the Bayesian approach for analysis, are illustrated in Figure 4. The results highlight the existence of potential numerical correlations among the main significant parameters. By considering all uncertainties in the data and the modeling as statistical distributions, the MCMC method offers a means to assess the dependability of the model parameters. The contour plots illustrating the results of parameter estimation show the interrelation between the parameters. The accumulation of the points in each part corresponds to the probability when the circle indicates the regions of the parameter with a 95% probability. The analysis of Figure 4 emphasizes that all parameters are appropriately defined and exhibit no correlation. The presence of well-centered round probability distributions in the projected distributions is proof of this.

## 4 Conclusion

Catalytic processes create a complex structure of reaction steps containing reactants, products, and intermediates. One of the complementing strategies proposed to describe the characteristics of complex kinetic networks is the reaction route approach, which provides a more comprehensive framework for understanding multistep reaction mechanisms, moving beyond the limitations of the single rate-determining step (RDS) concept. This approach is particularly valuable for reactions with multiple steps. Rather than simplifying complex mechanisms by focusing on a single RDS, the theory examines the intricate interplay between various steps and their collective impact on the overall reaction rate. Identifying basic reaction routes, and hence the comprehensive rate equation of any reactant/product, is a suitable method for analyzing the reaction pathways. A kinetic study employing complex reaction theory was conducted to investigate the oxidative desulfurization of DBT using UiO-66-NO_2_ at varying temperatures (20, 36, 60, 84, and 100 °C). Eleven elementary reactions were postulated in this study to describe the rates of formation or consumption of molecular components. In addition, the removal rate of DBT to the polar phase was based on the assumption of two lumped reactions. The kinetic parameters of DBT ODS reactions have been optimized, and the rate constants were determined through utilizing a simultaneous code connection to model the reactor with the MATLAB software. The activation energy and rate coefficients were determined by linear reparametrized Arrhenius plots. DBTO showed higher oxidation rate than DBT oxidation, DBT adsorption kinetic is greater than DBTO_2_ desorption. Ultimately, this investigation proposed a procedure for the integration of complex theory and multiple reactants, products, and surface step reactions. The model accurately identifies the kinetics of the reactions through the results obtained from parameter estimation. The kinetic parameters were adjusted to minimize error, resulting in an RMSE of 10.5%. All of the model parameters were identified with a 95% probability and were devoid of cross-correlation, according to the MCMC analysis.
